# Cancer is associated with severe disease in COVID-19 patients: a systematic review and meta-analysis

**DOI:** 10.3332/ecancer.2020.1047

**Published:** 2020-05-18

**Authors:** Richard Ofori-Asenso, Oyepeju Ogundipe, Akosua Adom Agyeman, Ken Lee Chin, Mohsen Mazidi, Zanfina Ademi, Marie Louise De Bruin, Danny Liew

**Affiliations:** 1 Department of Epidemiology and Preventive Medicine, Monash University, Melbourne, Victoria, VIC 3004, Australia; 2 Copenhagen Centre for Regulatory Science, Department of Pharmacy, University of Copenhagen, 2100 Copenhagen, Denmark; 3 Cardiac Renal and Vascular Associates PC, Jackson, MS 39204, USA; 4 Centre for Medicine Use and Safety, Monash Institute of Pharmaceutical Sciences, Monash University, Victoria, VIC 3052, Australia; 5 Melbourne Medical School, University of Melbourne, Victoria, VIC 3010, Australia; 6 Department of Twin Research and Genetic Epidemiology, Kings College London, London, SE1 7EH, UK

**Keywords:** COVID-19, coronavirus, SARS, pandemic

## Abstract

Cancer patients are vulnerable to complications of respiratory viruses. This systematic review and meta-analysis sought to examine the prevalence of cancer and its association with disease severity in patients with novel coronavirus disease 2019 (COVID-19). Searches were performed in MEDLINE, EMBASE and ScienceDirect from their inception until 28 April 2020. Severe disease was considered to encompass cases resulting in death or as defined by the primary study authors. Meta-analysis was performed using random-effect models. We included 20 studies involving 32,404 patients from China, the United Kingdom, the United States, Italy, Singapore, Thailand, France, India and South Korea. The pooled prevalence of cancer was 3.50% (95% confidence interval (CI) 1.70 to 5.80). The pooled prevalence was not moderated by study mean age, proportion of females or whether the study was conducted in/outside of China. Patients with cancer were more likely to experience severe COVID-19 disease compared to patients without cancer (pooled risk ratio 1.76, 95% CI 1.39 to 2.23). Our findings reiterate the need for additional precautionary measures to ensure that patients with cancer are not exposed to COVID-19, and if they become infected, extra attention should be provided to minimise their risk of adverse outcomes.

## Inroduction

The world is battling an immense threat from novel corona virus disease 2019 (COVID-19). As of 28 April 2020, more than 3.1 million confirmed COVID-19 cases had been reported from over 150 countries, among whom over 220,000 had died [1]. Most COVID-19-related deaths have been attributed to multiple organ failure in older or comorbid individuals [2].

A recent meta-analysis estimated that 2% of patients with COVID-19 had cancer [3]. However, the analysis included only data from China and did not evaluate the association of cancer with severe COVID-19 disease. Cancer patients may be more susceptible to COVID-19 than healthy individuals due to their high immunosuppressive burden caused by the cancer and anticancer treatments [4]. Improved understanding of the burden of cancer in COVID-19 patients may help to guide clinical management. Hence, in this study, we aimed to estimate the prevalence of cancer among COVID-19-infected patients as well as ascertain the association between cancer and disease severity.

## Methods

A systematic review was performed in accordance with the recommendations outlined in the PRISMA statement [5] and the Cochrane Handbook [6]. We searched MEDLINE, EMBASE and ScienceDirect using the terms ‘comorbidities’ or ‘clinical characteristics’ or ‘epidemiological’ and ‘COVID-19’ or ‘Coronavirus’ or ‘2019-nCoV’ or ‘SARS-CoV-2’ or ‘2019 novel coronavirus’ (Supplemental Table S1). The search was last updated on April 28, 2020. Further searches were also performed via the websites of the World Health Organization (WHO) and key public health institutions in some of the most affected countries (Supplementary Table S2) [1]. The reference lists of identified studies were also screened for additional papers. Two reviewers (O.O and R.O) performed article screening and any disagreements were resolved via consensus. Severe disease was considered to encompass cases resulting in death [7] or as defined by the study authors [7]. The quality of individual studies was evaluated using the Newcastle–Ottawa Scale (NOS) for non-randomized studies [8]. For each study, two reviewers (R.O and O.O) independently collected data, including author details, country (region), mean age, proportion of females, and data on prevalence and disease severity. We excluded studies based on family clusters, those focusing solely on deceased individuals and case series involving <10 patients or only children. Moreover, reviews, commentaries and editorials were excluded. Furthermore, because a national-based study in China was published with data up to February 11, 2020, we excluded all sub-national studies in China that recruited only patients up until that date. However, if a study based in China recruited patients beyond this date, they were included. Also, if studies from the same region or hospital recruiting patients over the period were present, we selected the report with the larger sample size or more detailed data.

Meta-analysis of prevalence was performed using Freeman–Tukey double arcsine transformation to adjust for variance instability [9]. Owing to anticipated between-study heterogeneity, random-effect model was used [6]. Furthermore, pooled risk ratios (RRs) and corresponding 95% confidence intervals (CIs) were derived to characterise the association between cancer and the occurrence of severe disease. Between-study heterogeneity was quantified using the *I*^2^ statistic [5]. A leave-one-out sensitivity analyses assessed the stability of pooled estimates. We applied meta-regression to determine whether the pooled prevalence was moderated by the age of study participants, gender distribution or location of the study (in/outside of China). All analyses were conducted using Stata SE software version 16 (StataCorp, TX, USA). A two-tailed *p*-value of <0.05 was considered as significant.

## Results

**Figure 1.**
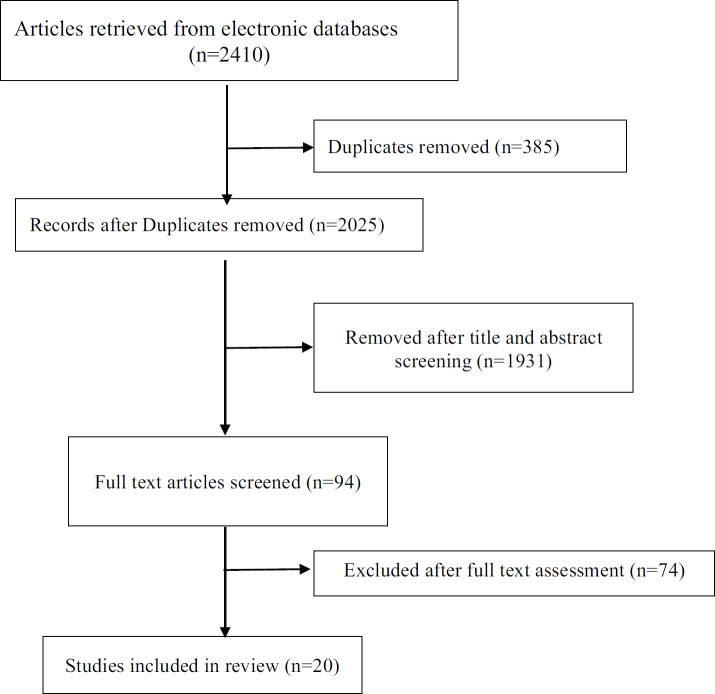
PRISMA flow chart of studies selection process.

The electronic searches retrieved 2,410 citations. Following removal of duplicates and screening of titles and abstracts, 94 articles were selected for full text evaluation. Twenty articles were retained after full-text assessment (Figure 1) [10–29]. The included studies were from China (*n* = 10), the United Kingdom (*n* = 2), South Korea (*n* = 1), the United States (*n* = 2), Italy (*n* = 1), Singapore (*n* = 1), India (*n* = 1), France (*n* = 1) and Thailand (*n* = 1). The studies involved a total of 32,404 patients. The mean age ranged from 40.3 to 75.0 years and 18.0% to 67.1% of the patients were females (Table 1).

**Figure 2.**
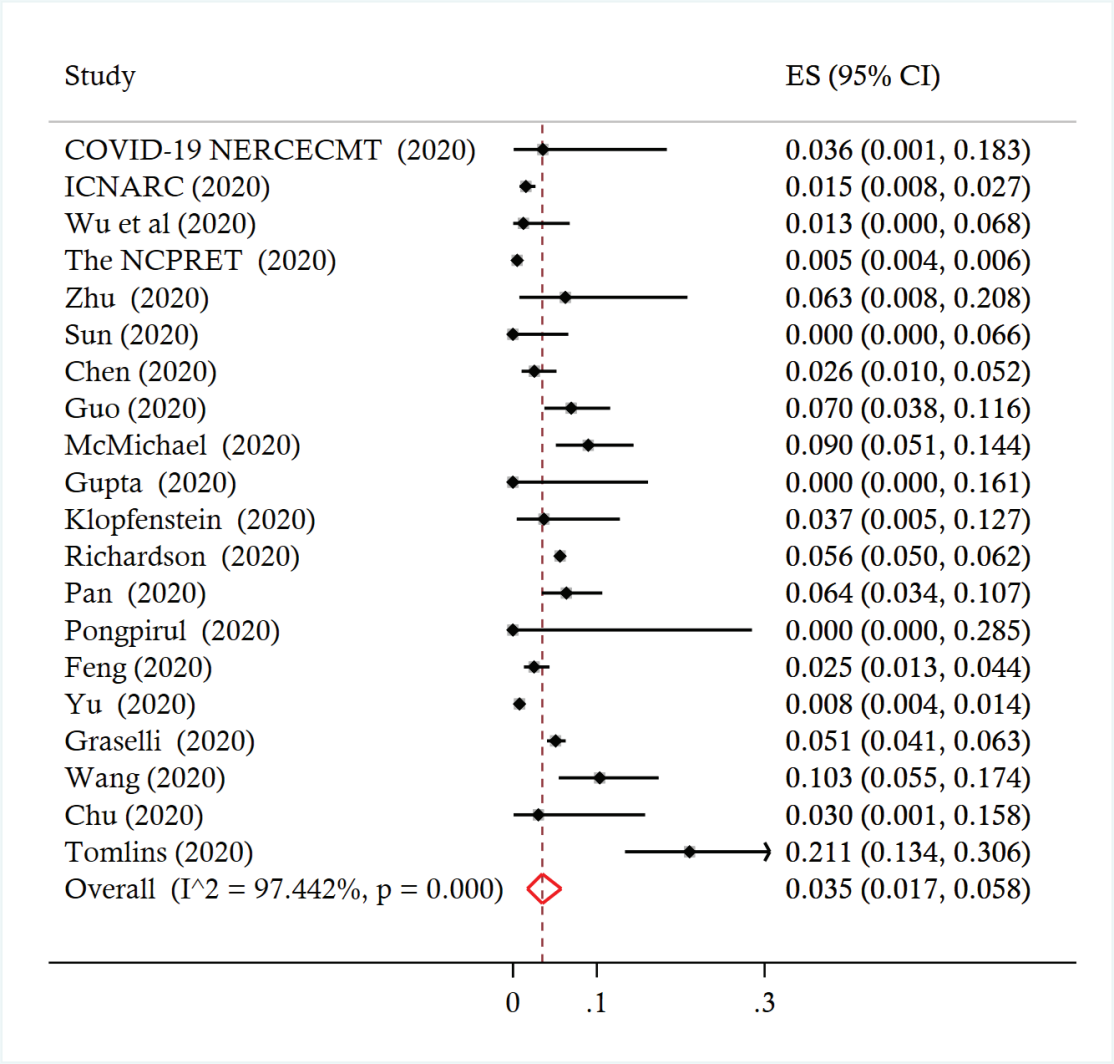
Forest plot of prevalence of cancer among patients infected with COVID-19.

**Figure 3.**
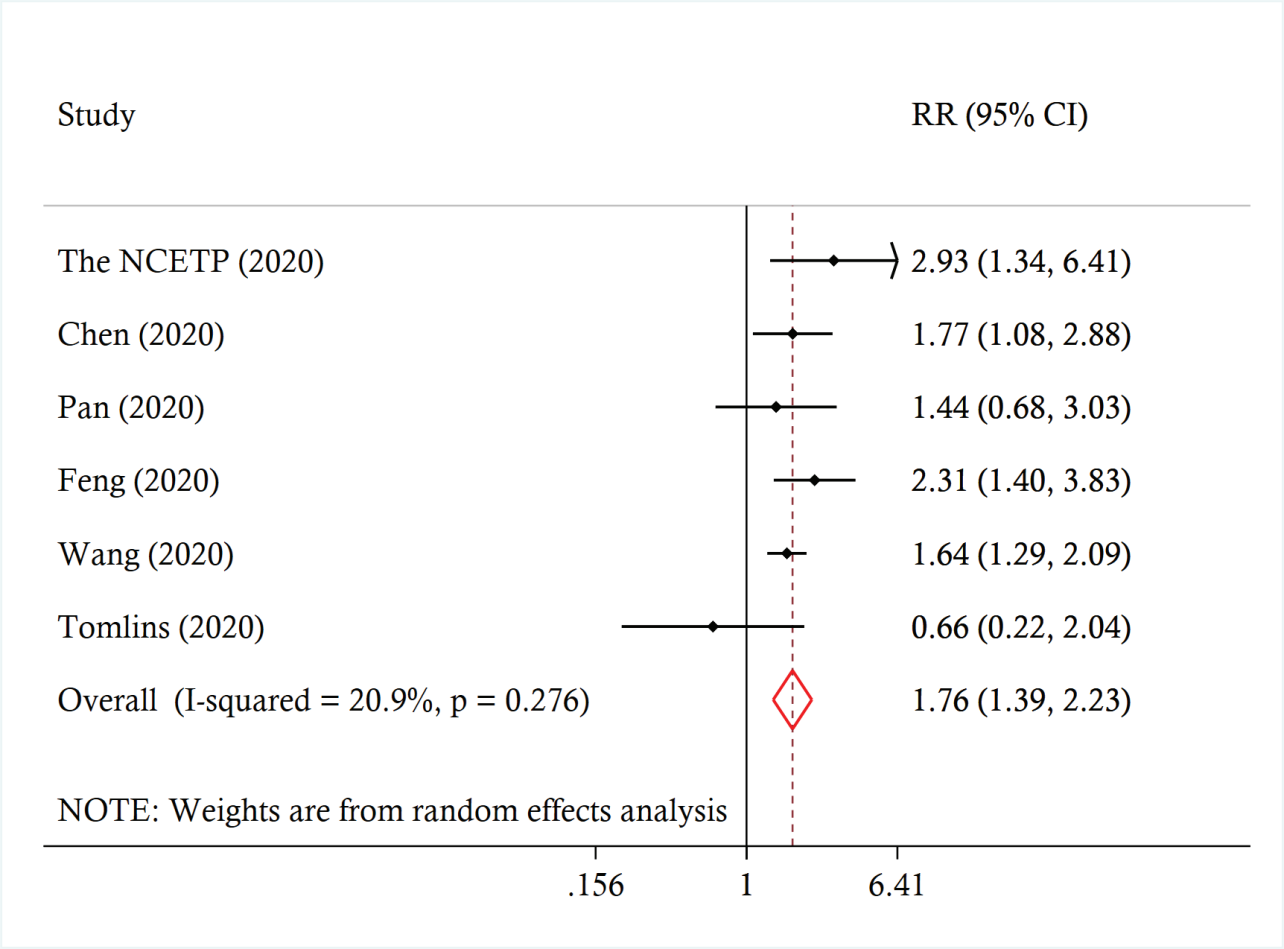
Forest plot of association between cancer and severe disease among COVID-19 patients.

Across the studies, the reported prevalence of cancer ranged from 0% to 21.0%. The pooled prevalence was 3.5% (95% CI 1.7 to 5.8%, *I*^2^ = 97.4%) (Figure 2). A leave-one-out sensitivity analysis did not change the results (point estimate ranged from 3% to 4%). There was no significant moderation of the pooled prevalence by the participants’ mean age (coefficient = 0.0034, *p* = 0.247), proportion of females (coefficient = 0.0006, *p* = 0.557) or being conducted in/outside of China (coefficient = −0.009, *p* = 0.795). Across six studies involving 22,046 patients, those with cancer were more likely to experience severe disease compared to patients without cancer (pooled risk ratio (RR_pooled_) 1.76, 95% CI 1.39 to 2.23, *I*^2^ = 20.9%) (Figure 3). A leave-one-out analyses did not change the results (the estimates ranged from RR_pooled_ 1.66 (95% CI 1.27 to 2.17) to 1.82 (95% CI 1.26 to 2.64)).

## Discussion

The results of our meta-analysis suggest a low prevalence of cancer among COVID-19 patients. However, patients with cancer are 76% more likely to experience severe disease compared to those without cancer. This finding is consistent with a recent study which reported that the fatality of cancer patients infected with the Middle East respiratory syndrome (MERS)-CoV in 2012 was significantly higher compared to patients without cancers [31]. The greater likelihood of severe COVID-19 in patients with cancer may reflect their increased vulnerability to developing complications of respiratory viruses [32]. Moreover, many oncology patients often have additional risk factors for severe COVID-19, such as advanced age and presence of other comorbidities [32].

Our study highlights the need to implement extra precautionary measures (including an awareness campaign) to ensure that patients with cancer are not exposed to the virus during the current outbreak and future outbreaks. The development of a COVID-19 vaccine or treatment modality may also be useful towards reducing the risk of this vulnerable population. Furthermore, it is imperative that during the current COVID-19 outbreak, measures are implemented to minimise interruptions in the provision of essential medical services to cancer patients.

Of note, as expected given the observational nature of the data, there was evidence of high statistical heterogeneity in the pooled prevalence of cancer. This does not necessarily invalidate the findings. Further assessment revealed that the heterogeneity was not entirely explained by differences in age or gender distribution among study population. Also, while the included studies originated from a few countries, around half were conducted in China which may affect the generalisability of our findings. Hence, further analysis would be necessary as the COVID-19 pandemic evolves and data from other regions become available.

## Conclusion

Our findings suggest that COVID-19 patients with cancer are more likely to experience severe disease than those without cancer. This emphasises the need to adopt additional precautionary measures to ensure that these vulnerable patients are not exposed to the virus, and if they become infected, extra attention should be provided to minimise their risk of adverse outcomes.

## Conflicts of interest

None.

## Funding

None.

## Figures and Tables

**Table S1.** Search strategy.

1 COVID-19.mp. 2 COVID-19.m_titl. 3 2019 novel coronavirus.mp. 4 2019 novel coronavirus.m_titl. 5 2019-nCoV.mp. 6 2019-nCoV.m_titl. 7 SARS-CoV-2.m_titl. 8 SARS-CoV-2.m_titl. 9 1 or 2 or 3 or 4 or 5 or 6 or 7 or 8 10 comorbidities.mp. or Comorbidity/ 11 comorbidity.mp. 12 clinical characteristics.mp. 13 clinical characteristics.m_titl. 14 epidemiological.mp. 15 epidemiolog*.mp. or Epidemiology/ 16 epidemiological.m_titl. 17 epidemiological.mp. 18 10 or 11 or 12 or 13 or 14 or 15 or 16 or 17 19 9 and 18

**Table S2.** Institutional websites of some of the countries searched.

Country	Institution	Website
Global	World Health Organization	https://www.who.int/
USA	Centers for Disease Control and Prevention	https://www.cdc.gov/
China	Chinese Centre for Disease Control and Prevention	http://www.chinacdc.cn/en/
Spain	National Centre of Epidemiology, Institute of Health Carlos III	https://www.isciii.es/Paginas/Inicio.aspx
Germany	Robert Koch Institute (RKI)	https://www.rki.de/EN/Home/homepage_node.html
France	Santé Publique France	https://www.santepubliquefrance.fr/
Iran	Ministry of Health and Medical Education	http://www.behdasht.gov.ir/
UK	Public Health England	https://www.gov.uk/government/organisations/public-health-england
Switzerland	Federal Office of Public Health	https://www.bag.admin.ch/bag/en/home.html
The Netherlands	National Institute of Public Health and the Environment	https://www.rivm.nl/en
Canada	Public Health Agency of Canada	https://www.canada.ca/en/public-health.html

**Table 1.** Characteristics of included studies.

Study No.	Author details**	Country (location)	Hospital	Last follow up	Sample size	Mean age	% Female	No. (%) with cancer	No. (%) severe disease^a^	
									Cancer patients	Non-cancer
1.	COVID-19 National Emergency Response Center, Epidemiology and Case Management Team, Korea Centers for Disease Control and Prevention [10]	South Korea	Korea Centers for Disease Control and Prevention	February 14, 2020	28	42.6	46.4	1 (3.6)	-	-
2.	ICNARC [11]	United Kingdom	England, Wales and Northern Ireland critical care units	March 26, 2020	775^a^	60.2	29.1	12 (1.2)	-	-
3.	Wu *et al* [12]	China (Jiangsu)	3 grade IIIA hospitals	February 14, 2020	80	46.1	51.3	1 (1.3)	-	-
4.	The Novel Coronavirus Pneumonia Emergency Response Epidemiology Team [13]	China	Nationwide	February 11, 2020	20982^b^	-	48.6	107 (0.5)	6 (5.6)	400 (1.9)
5.	Zhu *et al* [14]	China (Outside of Hubei)	Anhui Province ED	February 20, 2020	32	46.0	53.0	2 (6.3)	-	-
6.	Sun *et al* [15]	Singapore	National Centre for Infectious Diseases	February 16, 2020	54	42.0	46.3	0 (0.0)	-	-
7.	Chen *et al* [16]	China (Wuhan)	Tongji Hospital	February 28, 2020	274	62.0	38.0	7 (3.0)	5 (71.4)	108 (40.4)
8.	Guo *et al* [17]	China (Wuhan)	No. 7 Hospital of Wuhan	February 23, 2020	187	58.5	51.3	13 (7.0)	-	-
9.	McMichael *et al* [18]	US (Washington)	skilled nursing facility in King County	March 18, 2020	167	72.0	67.1	15 (9.0)	-	-
10.	Gupta *et al* [19]	India (New Delhi)	Sarfdarjung hospital	March 19, 2020	21	40.3	33.3	0 (0.0)	-	-
11.	Klopfenstein *et al* [20]	France	NFC hospital	March 17, 2020	54	47.0	67.0	2 (4.0)	-	-
12.	Richardson *et al* [21]	US (New York)	Northwell Health hospitals	April 4, 2020	5700	63.0	39.7	320 (6.0)	-	-
13.	Pan *et al* [22]	China (Hubei)	Wuhan Hanan Hospital, Wuhan Union Hospital, and Huanggang Central Hospital	March 18, 2020	204 (digestive =103)	52.9	52.5	13 (6.37)	4 (50.0)^c^	33 (34.7)^c^
14.	Pongpirul *et al* [23]	Thailand	Bamrasnaradura Infectious Diseases Institute	January 31, 2020	11	61.0	45.5	0 (0)	-	-
15.	Feng *et al* [24]	China (Multicentre)	Jinyintan Hospital in Wuhan, Shanghai Public Health Clinical Center in Shanghai and Tongling People’s Hospital in Anhui Province	February 15, 2020	476	53.0	43.1	12 (2.5)	7 (58.3)	117 (25.2)
16.	Yu *et al* [5]	China (Wuhan)	Zhongnan Hospital of Wuhan University	February 17, 2020	1524	-	-	12 (0.79)	-	-
17.	Grasselli *et al* [26]	Italy (Lombardy)	72 hospitals	March 18, 2020	1591^a^	63.0	18.0	81 (8.0)	-	-
18.	Wang et al [27]	China (Wuhan)	Renmin Hospital of Wuhan University	February 13, 2020	116	54	42.2	12 (10.3)	11 (91.6)	58/104
19.	Chu *et al* [28]	China (Wenzhou city)	First Affiliated Hospital of Zhejiang University	February 23, 2020	33^a^	65.2	33.3	1 (3.0)	-	-
20.	Tomlins *et al* [29]	UK (England)	North Bristol NHS Trust	March 30, 2020	95	75.0	37.0	20 (21)	3 (15.0)	17 (22.7)
